# Treatment outcomes and considerations for topical immunotherapy in patients with alopecia totalis and alopecia universalis

**DOI:** 10.3389/fmed.2025.1573929

**Published:** 2025-07-25

**Authors:** Taiyo Hitaka, Sanehito Haruyama, Shun Ohmori, Natsuko Saito-Sasaki, Etsuko Okada, Motonobu Nakamura, Yu Sawada

**Affiliations:** ^1^Department of Dermatology, University of Occupational and Environmental Health, Kitakyushu, Japan; ^2^Haruyama Dermatology Clinic, Gyota City, Japan; ^3^Department of Dermatology, Kokura Daiichi Hospital, Kokura City, Japan; ^4^Department of Dermatology, Takasaki General Medical Center, Takasaki, Japan; ^5^Matsuoka Clinic, Osaka, Japan

**Keywords:** alopecia totalis, alopecia universalis, topical immunotherapy, SADBE, DPCP

## Abstract

**Background:**

Alopecia areata (AA) is a chronic immune-mediated disorder causing non-scarring hair loss. Severe forms like alopecia totalis (AT) and alopecia universalis (AU) pose therapeutic difficult situations. Topical immunotherapy with squaric acid dibutylester (SADBE) and diphenylcyclopropenone (DPCP) is widely used but has variable efficacy.

**Objectives:**

To evaluate the efficacy of topical immunotherapy in severe AA, identify factors influencing outcomes, and assess adjunctive antihistamine therapy.

**Methods:**

A retrospective analysis of 106 severe AA cases (26 AT, 80 AU) among 1,098 patients diagnosed between 2007 and 2016. Treatment efficacy was assessed using the Alopecia Areata Investigational Assessment Guidelines (AAIAG), and statistical analyses included chi-square tests and Kaplan-Meier analysis.

**Results:**

Among the 106 patients included in this study, 43% exhibited excellent or good responses to topical immunotherapy, while 75% experienced at least partial hair regrowth. Patients with alopecia totalis demonstrated slightly better outcomes than those with alopecia universalis (50% vs. 40% achieving excellent or good responses). Atopic dermatitis was significantly associated with lower treatment efficacy (54% vs. 80%, *p* = 0.0157). Although antihistamine use showed a trend toward improved responses (78% vs. 38%), the difference did not reach statistical significance (*p* = 0.0991), and multivariate analysis did not confirm its efficacy (*p* = 0.649). Hair regrowth was observed within 4 months in 90% of cases, while peak therapeutic effects were achieved within 3 years. Long-term treatment adherence correlated with improved outcomes, highlighting the importance of sustained therapy.

**Conclusion:**

Topical immunotherapy remains an effective treatment for severe alopecia areata, although response rates vary among patients. Individualized treatment approaches, including prolonged therapy and consideration of patient-specific factors, are essential for optimizing clinical outcomes.

## Introduction

Alopecia areata (AA) is a chronic, immune-mediated disorder that manifests as non-scarring hair loss, affecting the scalp and other body areas (1, 2). The precise etiology of AA remains elusive, but it is believed to involve autoimmune mechanisms targeting hair follicles in the anagen phase (3). Clinically, AA presents as a spectrum ranging from localized hair loss to severe forms such as alopecia totalis (AT) and alopecia universalis (AU), which are characterized by extensive or complete hair loss (4, 5). These severe forms often pose substantial therapeutic challenges and are frequently associated with comorbidities, particularly atopic dermatitis.

Several treatment options have been proposed for severe alopecia areata (AA), including corticosteroids (6, 7), topical immunotherapy (8, 9), and more recently, Janus kinase (JAK) inhibitors (10, 11). Among them, topical immunotherapy using sensitizing agents such as squaric acid dibutylester (SADBE) and diphenylcyclopropenone (DPCP) has long been used as a one of the first-line therapies (8, 9, 12, 13), particularly in situations where systemic immunosuppressants or JAK inhibitors are unavailable, contraindicated, or less accessible due to cost or safety concerns. Therefore, this study focuses on evaluating the efficacy and influencing factors of topical immunotherapy in patients with severe forms of AA.

Its mechanism of action is thought to involve the suppression of pathogenic T-cell activity and modulation of cytokine production, promoting an immunomodulatory environment around the affected hair follicles (14). Despite its wide adoption, the efficacy of topical immunotherapy remains inconsistent (15). In addition, the variability in response rates, particularly among patients with severe forms like AT and AU, has not been fully explained. Additionally, while atopic dermatitis has been identified as a factor that negatively impacts treatment efficacy, the underlying mechanisms and potential strategies to mitigate this effect remain unclear (16). Furthermore, the potential for adjunctive therapies, such as antihistamine administration, to enhance the efficacy of topical immunotherapy warrants further investigation (17).

This study aims to address these critical gaps by conducting a retrospective analysis of patients diagnosed with AA, focusing on those with severe cases of AT and AU. The primary objectives of this research are threefold: (1) To evaluate the overall efficacy of topical immunotherapy in a large cohort of severe AA cases; (2) to identify patient-specific factors, including comorbid atopic dermatitis, that influence treatment outcomes; and (3) to assess the potential role of adjunctive antihistamine therapy in enhancing treatment efficacy. By systematically addressing these questions, this study seeks to advance the understanding of topical immunotherapy in severe AA and guide clinicians toward more effective, individualized treatment strategies.

## Materials and methods

### Patients

We conducted a retrospective analysis of medical records from 1,098 patients diagnosed with alopecia areata (AA) at our department between January 2007 and December 2016. Among them, patients with a fixed alopecia extent of 25% or more, as determined by the SALT score (18), were initially selected. From this group, only patients diagnosed with alopecia totalis (AT) or alopecia universalis (AU) were included in the final analysis, in order to focus on severe forms of AA. Patients were excluded in cases where they had received topical immunotherapy within 3 months of steroid pulse therapy, had less than 6 months of follow-up, or showed clear effects from other treatments.

The characteristics of patients, including gender, age (categorized as 20 years or younger versus older than 20 years), disease duration prior to treatment initiation, presence of atopic dermatitis at the time of treatment initiation, duration of topical immunotherapy, and reported adverse events during treatment, were comprehensively analyzed. Additionally, the types of alopecia were classified into standard alopecia areata, multiple alopecia areata, alopecia totalis, alopecia universalis, ophiasis alopecia areata, and other less common forms of alopecia. Information regarding antihistamine use was retrospectively collected from electronic medical records, based on prescription history at the time of treatment initiation.

### Study design and data collection

This study was conducted as a retrospective observational study. Data were extracted from electronic medical records and included clinical histories, physical examinations, and photographic documentation. Baseline characteristics, including patient demographics and alopecia severity at the initial visit, were examined.

### Evaluation criteria

**FIGURE 1:**
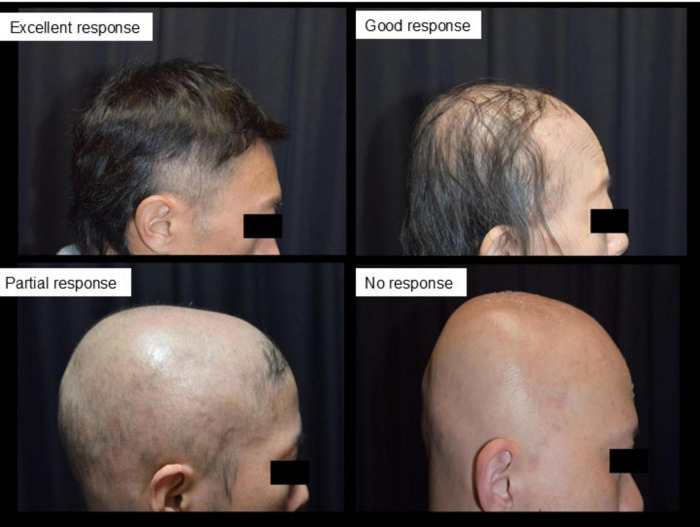
Evaluation criteria for treatment outcomes based on alopecia areata investigational assessment guidelines (AAIAG). This figure illustrates the classification criteria used to evaluate treatment efficacy in patients with severe alopecia areata. Responses were categorized into four levels: excellent (> 75% regrowth), good (50%–74% regrowth), partial (25%–49% regrowth), and no response (< 25% regrowth). These categories were determined based on clinical assessment and photographic documentation of hair regrowth.

Treatment outcomes were assessed using the Alopecia Areata Investigational Assessment (AAIAG) guidelines, which categorized responses into four levels: excellent (> 75% regrowth), good (50%–74% regrowth), partial (25%–49% regrowth), or no response (< 25% regrowth) (18) (Figure 1). The time to initial hair regrowth and the time to maximum observed regrowth were recorded for each patient.

### Topical immunotherapy

Topical immunotherapy was conducted by applying squaric acid dibutyl ester (SADBE) or diphenylcyclopropenone (DPCP) once every 1–4 weeks. Treatment procedures were standardized, with the concentration of the applied agent adjusted according to patient tolerance and clinical response. Clinical photographs were compared for each case to evaluate efficacy.

### Statistical analysis

Descriptive statistics were used to summarize patient demographics and treatment outcomes. Chi-square tests were applied to assess the association between patient characteristics and treatment efficacy. A *p*-value of < 0.05 was considered statistically significant. Additionally, univariate and multivariate logistic regression analyses were performed to identify independent predictors of treatment response. Odds ratios (OR) and 95% confidence intervals (CI) were calculated to evaluate the strength of associations. All statistical analyses, including descriptive statistics, group comparisons, and regression analyses, were performed using IBM SPSS Statistics version 27 (IBM, Chicago, IL) and GraphPad Prism version 9.5.0 (Graphpad Software, San Diego, CA). Statistical analyses were conducted using appropriate software, and all tests were two-tailed with a significance level set at *p* < 0.05.

### Ethical considerations

This study was approved by the Institutional Review Board of our institution. The requirement for informed consent was waived due to the retrospective nature of the study. All patient data were anonymized and handled in compliance with ethical guidelines to ensure confidentiality and privacy.

## Results

### Patient background

To better understand the characteristics of the study cohort, we first analyzed the demographic and clinical profiles of the included patients. Among the 1,098 patients diagnosed with alopecia areata at our department, the breakdown of alopecia types revealed a diverse spectrum (Table 1). The distribution of alopecia types was as follows: standard alopecia areata (159 cases), multiple alopecia areata (622 cases), alopecia totalis (46 cases), alopecia universalis (180 cases), ophiasis alopecia areata (14 cases), and other types (77 cases). These categories represent the initial cohort before stratification. For the main analyses, we focused exclusively on patients with alopecia totalis and universalis. Among these, 106 patients met the inclusion criteria for this study, comprising 26 cases of alopecia totalis and 80 cases of alopecia universalis.

**TABLE 1:** Types of alopecia in 1,098 patients seen in our department.

Types of alopecia	Number of cases
Standard AA	159 cases
Multiple AA	622 cases
Alopecia totalis	46 cases
Alopecia universalis	180 cases
Ophiasis AA	14 cases
Other types of alopecia	77 cases
**Total**	**1098 cases**

Demographically, the study population included 44 males and 62 females, highlighting a slight predominance of female patients. Patient ages ranged widely from 1 to 91 years, with a median age of 33 years. Disease duration prior to treatment initiation varied extensively, spanning from 1 to 612 months, with a median duration of 18 months. The duration of topical immunotherapy ranged from 3 to 125 months, with a median of 24 months. Notably, 22 patients had clinically diagnosed atopic dermatitis at the time of treatment initiation, which is a known factor that may negatively influence the outcomes of topical immunotherapy (15). Nine patients experienced adverse events, including autosensitization dermatitis (Table 2). No patients discontinued treatment due to adverse effects.

**TABLE 2:** The patient characteristics of this study included 26 cases of alopecia totalis and 80 cases of alopecia universalis.

Variables		
Age	1-91	Median: 33
Gender		
Male	44 cases	
Female	62 cases	
Disease duration	1-612 months	Median 18 months
Duration of topical immunotherapy	3-125 Months	Median 24 months
Cases with atopic dermatitis	22 cases	
Adverse events		
Autosensitization dermatitis	9 cases	

### Treatment efficacy

**FIGURE 2:**
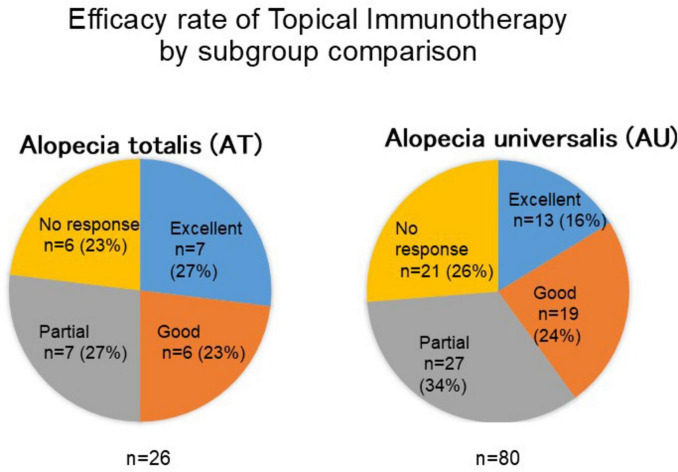
Comparison of treatment outcomes between alopecia totalis and alopecia universalis. This figure presents the response rates to topical immunotherapy in patients with alopecia totalis (AT) and alopecia universalis (AU). The proportion of patients achieving excellent or good responses was higher in the AT group (50%) compared to the AU group (40%), suggesting that baseline hair loss extent may influence treatment efficacy.

The therapeutic efficacy of topical immunotherapy was evaluated by analyzing hair regrowth outcomes. A subgroup comparison revealed that patients with alopecia totalis achieved slightly better outcomes, with 50.0% showing excellent or good responses compared to 40.0% of patients with alopecia universalis (Figure 2). This finding suggests that the extent of baseline hair loss may influence treatment outcomes.

**FIGURE 3:**
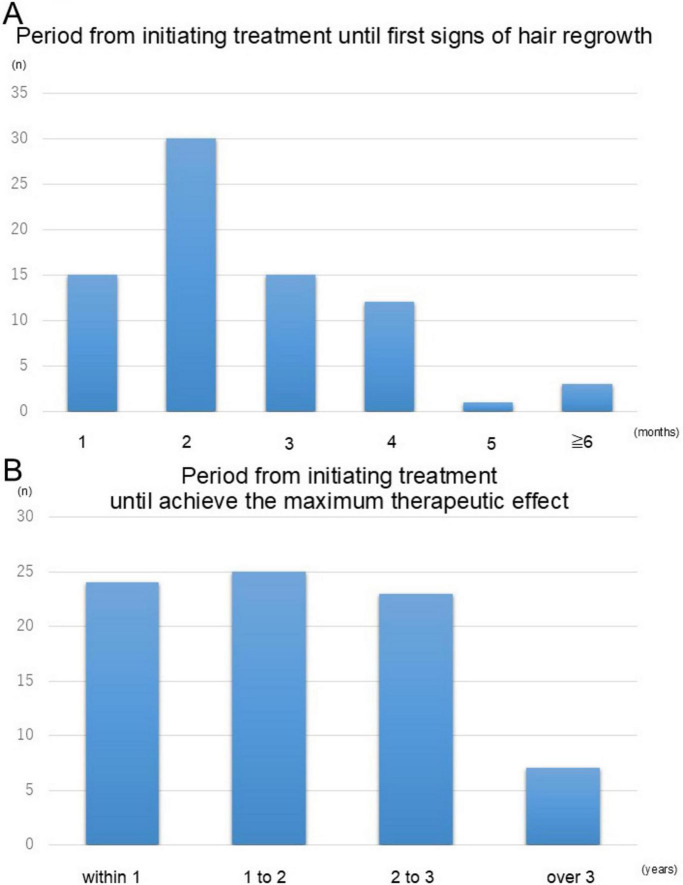
Timeline of hair regrowth following topical immunotherapy. (A) The proportion of patients exhibiting initial signs of hair regrowth over time. The majority (94.7%) experienced regrowth within 4 months of treatment initiation. (B) The time required to achieve the maximum therapeutic effect. Most patients (91.3%) reached their peak response within 3 years of treatment initiation, with a substantial number achieving this outcome between 1 and 2 years. These findings emphasize the importance of sustained therapy for optimal outcomes.

We next analyzed the temporal aspects of hair regrowth to understand the timeline of response to topical immunotherapy. Among the cases with observed hair regrowth, excluding those with no change or worsening, 94.7% experienced their first signs of regrowth within 4 months of initiating treatment (Figure 3A). The longest time to initial regrowth recorded was 1 year and one case discontinued treatment after only 3 months due to insufficient efficacy. Further analysis of the time required to achieve the maximum therapeutic effect revealed that 91.3% of patients reached their peak response within 3 years of treatment initiation, with a substantial proportion achieving this milestone between 1 and 2 years (Figure 3B). These findings highlight the importance of consistent and prolonged therapy to maximize treatment outcomes in patients with severe cases of alopecia areata.

### Influence of various conditions on treatment efficacy

The impact of patient-specific factors on the efficacy of topical immunotherapy was systematically evaluated. Patients were stratified based on gender, age, disease duration, and the presence of atopic dermatitis. Statistical analyses revealed no significant differences in response rates between genders, age, and disease duration (Table 3).

**TABLE 3:** Comparison of the efficacy of topical immunotherapy based on various conditions.

Variables		Number of cases	Effective or better response	Unchanged/Worsened	*P*-value
Gender	Male	44	34	10	0.5848
	Female	62	45	17	
Age	<20	23	16	7	0.537
	≥20	83	63	20	
Disease duration	<1 year	43	34	9	0.3753
	≥1 year	63	45	18	
Atopic dermatitis	Presence	22	12	10	0.0157
	Absence	84	67	17	

However, patients with atopic dermatitis demonstrated significantly lower response rates compared to those without cases. To further investigate this observation, we analyzed the influence of antihistamine usage in patients with atopic dermatitis. Although the difference was not statistically significant (*p* = 0.0991), patients receiving antihistamine therapy showed a higher response rate (78%) compared to those who did not (38%) (Table 4). This trend suggests a possible potential benefit of adjunctive antihistamine therapy in enhancing the effectiveness of topical immunotherapy for patients with comorbid atopic dermatitis.

**TABLE 4:** Comparison of the efficacy of topical immunotherapy based on antihistamine usage in patients with atopic dermatitis.

Anti-histamine drug usage	Total number	Effective or Better response	Unchanged/worsened	*P*-value
+	9	7	2	0.0991
-	13	5	8	

### Influence of various conditions on treatment efficacy

To further evaluate the impact of patient-specific factors on the efficacy of topical immunotherapy, we conducted univariate and multivariate analyses (Table 5). The results of the univariate analysis indicated that age (OR = 0.9, 95% CI: 0.4–2.2, *p* = 0.835), gender (OR = 0.6, 95% CI: 0.2–1.4, *p* = 0.209), and disease duration (OR = 1.5, 95% CI: 0.6–3.7, *p* = 0.354) were not significantly associated with treatment response. However, the presence of atopic dermatitis was found to be a significant predictor of poor response (OR = 3.3, 95% CI: 1.2–8.9, *p* = 0.019). In contrast, antihistamine use did not show a significant association with treatment efficacy (OR = 0.5, 95% CI: 0.2–1.4, *p* = 0.192).

**TABLE 5:** Univariate and multivariate analysis.

Variables	Univariate analysis		Multivariate analysis	
	Odds ratio	*P*-value	Odds ratio	*P*-value
Age	0.9 (0.4-2.2)	0.835	1.8 (0.57-5.5)	0.323
Gende	0.6 (0.2-1.4)	0.209	0.6 (0.2-1.6)	0.346
Duration	1.5 (0.6-3.7)	0.354	1.6 (0.6-4.2)	0.320
Presence of Atopic dermatitis	3.3 (1.2-8.9)	0.019	4.6 (1.3-16.4)	0.020
Antihistamine use	0.5 (0.2-1.4)	0.192	0.8 (0.3-2.3)	0.649

Multivariate analysis further reinforced these findings. Age (OR = 1.8, 95% CI: 0.57–5.5, *p* = 0.323), gender (OR = 0.6, 95% CI: 0.2–1.6, *p* = 0.346), and disease duration (OR = 1.6, 95% CI: 0.6–4.2, *p* = 0.320) remained non-significant predictors of treatment outcomes. However, the presence of atopic dermatitis continued to be a significant negative factor affecting response rates (OR = 4.6, 95% CI: 1.3–16.4, *p* = 0.020). On the other hand, antihistamine use did not demonstrate a statistically significant association with treatment outcomes (OR = 0.8, 95% CI: 0.3–2.3, *p* = 0.649). These findings suggest that while patient demographics and disease duration do not significantly influence the efficacy of topical immunotherapy, the presence of atopic dermatitis is associated with poorer treatment outcomes.

## Discussion

Topical immunotherapy remains a widely used and accessible treatment for severe AA. While newer therapies such as JAK inhibitors have emerged, topical immunotherapy continues to play an important role, particularly in long-term management and resource-limited situations. In alopecia areata guidelines in various countries (19–22), topical immunotherapy is recommended. This therapy is considered a first-line option for cases where fixed alopecia patches cover 25% or more of the scalp, such as in severe cases of alopecia totalis and alopecia universalis. Previous meta-analyses reported that 42.6% of patients achieved full regrowth, while 56.1% experienced substantial regrowth defined as more than 70% (23). In the present study, the proportion of patients achieving effective responses (defined as regrowth on ≥ 25% of the scalp surface) was 43%, slightly lower than previous studies. However, 75% of cases demonstrated some degree of regrowth included partial responses cases. These findings suggest that topical immunotherapy remains a valuable treatment option even for severe cases, with consistent long-term treatment playing a key role in achieving better outcomes.

Analysis of the time to achieve treatment effects indicated that hair regrowth was typically observed within 6 months, even in cases where early response was limited. Furthermore, it was shown that maximum therapeutic outcomes often required treatment durations of up to 3 years. These results underscore the importance of perseverance in continuing therapy for patients with severe alopecia areata.

Patients with alopecia areata frequently have atopic predispositions, and previous studies have suggested higher rates of atopic dermatitis among those with severe alopecia. However, the precise relationship between atopic dermatitis and alopecia areata remains unclear, necessitating further investigation. Our findings also revealed that patients with atopic dermatitis showed significantly lower response rates to topical immunotherapy compared to those without atopy. This aligns with prior studies suggesting that atopic dermatitis may hinder treatment efficacy.

Additionally, antihistamine co-administration in patients with atopic dermatitis has been reported to enhance the efficacy of topical immunotherapy by reducing IFN-γ production, ICAM-1 expression in the skin, and substance P levels (24). Although our study did not demonstrate a statistically significant benefit, patients receiving antihistamines showed a trend toward higher response rates (78% vs. 38%). This suggests a potential synergistic effect between antihistamines and topical immunotherapy, even in severe cases such as alopecia totalis and alopecia universalis. However, the observed difference did not reach statistical significance (*p* = 0.0991), and multivariate analysis did not support its efficacy (*p* = 0.649). These discrepancies may be influenced by unmeasured confounding factors or patient-specific characteristics. Further studies with larger cohorts and a more comprehensive patient stratification are needed to validate these findings.

A limitation of this study is the lack of detailed information on the severity of atopic dermatitis. As severity was not systematically recorded in the medical charts, stratified analysis based on AD severity could not be performed. Another limitation of this study is the lack of detailed information regarding the types and dosages of anti-histamines used. Although a trend toward improved outcomes was observed in patients who used anti-histamines, this result should be interpreted cautiously. In this study, we could not determine the specific conditions for which anti-histamines were administered. It is therefore difficult to draw conclusions regarding the indications or mechanisms underlying the potential benefits observed. Further investigation focusing on specific diseases or indications for antihistamine use may help clarify these associations. A further limitation of this study is that precise SALT scores at follow-up were not consistently documented. As treatment response was mainly evaluated through photographic comparison, the absence of standardized quantitative assessment may limit the accuracy and objectivity of the efficacy evaluation.

Although pairwise comparisons showed a statistically significant difference between the SADBE-only group and the switching group (Tables 6, 7), this result should be interpreted cautiously. The switching group consisted of patients primarily due to insufficient efficacy, the precise reasons for switching, such as lack of efficacy versus adverse events, could not be fully determined from the available records. In addition, the frequency of topical immunotherapy application was not consistently analyzed, making it difficult to assess the impact of treatment intervals on outcomes. These limitations should be taken into account interpreting the results.

**TABLE 6:** Summary of treatment efficacy for DPCP, SADBE, and Combined Use.

Efficacy	DPCP only	SADBE only	Switching
Excellent	2	17	1
Good	2	19	4
Partial	3	26	5
No response	1	7	19

**TABLE 7:** Pairwise comparison of treatment efficacy with Bonferroni correction.

Pairwise comparison	Unadjusted *p*-value	Bonferroni-adjusted *p*-value
DPCP only vs SADBE only	1.00	1.00
DPCP only vs switching	0.04	0.11
SADBE only vs switching	<0.01	<0.01

In conclusion, this study reaffirms the role of topical immunotherapy as an effective treatment for severe alopecia areata. While response rates may vary based on patient-specific factors such as the presence of atopic dermatitis, the potential for adjunctive therapies like antihistamines offers a promising avenue for enhancing outcomes. Continued research and accumulation of clinical evidence will further refine treatment strategies and improve patient care.

## Data Availability

The raw data supporting the conclusions of this article will be made available by the authors, without undue reservation.
